# Interleukin-2 is a potent latency reversal agent in people with treated HIV-1

**DOI:** 10.1126/sciadv.aea4268

**Published:** 2025-12-19

**Authors:** Michael L. Freeman, Brian M. Clagett, Konstantin Leskov, Daniela Moisi, George A. Yendewa, Scott F. Sieg, Gregory M. Laird, Jonathan Karn, Jeffrey M. Jacobson, Benigno Rodriguez, Michael M. Lederman

**Affiliations:** ^1^ Rustbelt Center for AIDS Research, Case Western Reserve University, Cleveland, OH, USA.; ^2^ Division of Infectious Diseases and HIV Medicine, Department of Medicine, Case Western Reserve University/University Hospitals Cleveland Medical Center, Cleveland, OH, USA.; ^3^ Center for Global Health and Diseases, Department of Pathology, Case Western Reserve University, Cleveland, OH, USA.; ^4^ Department of Molecular Biology and Microbiology, Case Western Reserve University, Cleveland, OH, USA.; ^5^ Accelevir Diagnostics, Baltimore, MD, USA.

## Abstract

Eliciting HIV-1 expression from latently infected CD4^+^ T cells allows these rare reservoir cells to become targets of the immune system and may also result in death of reservoir cells via virus-induced cytopathy. We asked whether administration of recombinant interleukin-2 (rIL-2) to people with HIV-1 (PWH) on antiretroviral therapy (ART) would induce HIV-1 replication and activate cellular immunity. Nine men with ART-suppressed HIV-1 completed a single 4-day cycle of rIL-2 administration. Plasma HIV-1 RNA levels rose from <20 copies per milliliter at entry to a mean of 301 copies per milliliter at day 7 (an average increase of 0.82 log_10_; *P* = 0.008). In addition, we observed robust natural killer and T cell activation, with a modest increase in regulatory T cell–like cells. Thus, rIL-2 appears to be one of the most potent latency-reversing agents tested in PWH on ART.

## Teaser

Recombinant IL-2 induces robust HIV-1 reactivation and immune activation in people with HIV-1 on antiretroviral therapy.

## INTRODUCTION

Replication-competent HIV-1 can be isolated from latently infected, resting CD4^+^ T cells (the “viral reservoir”) in persons with HIV-1 (PWH) (*1*–*3*). While combination antiretroviral therapy (ART) often controls HIV-1 replication to levels below limits of detection, ART must be taken life-long despite its metabolic and cardiovascular toxicities and substantial cost. Latently infected CD4^+^ T cells, which are largely invisible to immune surveillance, constitute the principal impediment to the goal of finding a scalable intervention that eliminates or permanently neutralizes HIV-1 in the absence of ART (*4*).

One strategy to eliminate the viral reservoir is the “shock and kill” approach in which latency reversal agents (LRAs) shock latently infected CD4^+^ T cells into reactivating viral expression, thereby subjecting them to both cytopathic effects of virus replication and cytolytic targeting by the immune system (the kill) (*5*). Many compounds are capable of eliciting expression of HIV from latently infected CD4^+^ T cells in vitro, but few have shown efficacy at reducing the latent HIV-1 reservoir in vivo. Absent additional immune activation and effector mechanisms, the cytopathic effect of HIV replication upon reactivation is insufficient to reduce the viral reservoir.

There is substantial evidence that recombinant interleukin (IL-2) (rIL-2) administration in PWH could both stimulate HIV reactivation in vivo and provide an immunologic boost that might target the viral reservoir. Aldesleukin, an rIL-2 with the trade name Proleukin, is a potent immunomodulator currently licensed for treating and demonstrated to enhance tumor regression and tumor-specific T cell activation in metastatic renal cell carcinoma and metastatic melanoma (*6*). In vitro, IL-2 elicits HIV-1 expression from CD4^+^ T cells (*7*) and promotes natural killer (NK) and T cell activation and proliferation (*8*). These effects are largely driven by IL-2–induced Janus kinase (JAK) signal transducer and activator of transcription (STAT) pathway activation, which leads to STAT5 phosphorylation (pSTAT5) (*9*). Notably, the long terminal repeat region of HIV has several pSTAT5-binding sites (*10*), and blocking pSTAT5 with the JAK inhibitors ruxolitinib or tofacitinib impaired HIV-1 replication ex vivo and in vitro (*11*). IL-2 has also been extensively studied in PWH in vivo and has been consistently shown to increase circulating CD4^+^ T cell numbers (*12*–*15*). Despite this clear effect, rIL-2 administration demonstrated no clinical benefits regarding improving survival or decreasing risk of progression to AIDS in two landmark, international, multicenter, phase 3, randomized trials of rIL-2 in PWH (*16*, *17*).

Notably, IL-2 can elicit HIV-1 expression from CD4^+^ T cells in vitro (*7*), and several early studies of IL-2 administration in viremic PWH found that plasma viral load levels transiently increased following cycles of IL-2 (*18*, *19*). In addition, in a nonrandomized, cross-sectional study comparing 14 PWH receiving a modern potent ART regimen plus IL-2 versus 12 receiving ART alone, Chun and colleagues (*12*) found that replication-competent virus could be isolated from resting CD4^+^ T cells in only 8 of 14 participants who received rIL-2 plus ART (versus 12 of 12 who received ART alone), suggesting a lower pool of CD4^+^ T cells containing replication-competent HIV-1 in rIL-2 recipients. Thus, we decided in this study to ask whether IL-2 administration could induce virus expression in persons with ART-controlled HIV-1 and reduce the HIV-1 latent reservoir, thereby establishing rIL-2 as a therapy to be used in conjunction with other approaches to cure HIV-1.

## RESULTS

Nine men with HIV-1 were enrolled {median age [interquartile range (IQR)]: 50 years (38 to 56); three were African American/Black and six were white}, all of whom had ART-mediated virus suppression (all ≤34 copies plasma HIV-1 RNA/ml at screening, six of nine without detectable HIV-1 RNA) and robust CD4^+^ T cell counts [median (IQR): 725 cells/μl (612 to 1017) at screening]. All received a single 4-day cycle of twice-daily subcutaneous (sc) rIL-2 administration [5 million international units (MIU) per dose], and some received additional cycles, 8 weeks apart. This dose and schedule of rIL-2 administration were selected as they were comparable to dosing used in the landmark SILCAAT (4.5 MIU twice-daily for six cycles) and ESPRIT (7.5 MIU twice-daily for three cycles) studies and other trials of sc rIL-2 (*16*, *20*). The dose of rIL-2 used in our study can be considered a low-dose treatment relative to the dose of rIL-2 FDA-approved to treat metastatic renal cell carcinoma or metastatic melanoma. Although the first cycle was uniformly safe without serious adverse events, the study was terminated in consultation with the external safety monitoring committee because of toxicities that ultimately developed in three participants: One had capillary leak syndrome requiring hospitalization, and two had reversible biochemical hypothyroidism (Table 1). The capillary leak syndrome resolved as did the transient hypothyroidism that was associated with elevated levels of antibody to thyroid peroxidase.

**Table 1.** Participant characteristics.

Participant ID	Age	Race	Sex	CD4^+^ T cell count per milliliter at screening	CD8^+^ T cell count per milliliter at screening	HIV-1 RNA copies per milliliter at screening	ART regimen at screening	Time on ART (year)	Number of rIL-2 treatment cycles	Serious adverse events
01	64	White	Male	725	451	Not detected	Genvoya	16.04	1	
02	59	White	Male	612	408	20	Triumeq, Prinzide	25.78	1	
03	43	White	Male	946	1121	Not detected	Descovy, Tivicay	9.10	3	
04	56	White	Male	1017	862	Not detected	Triumeq	24.94	3	Capillary leak syndrome
05	56	White	Male	478	302	21	Genvoya	9.76	3	Reversible biochemical hypothyroidism
06	38	African American/Black	Male	1078	842	Not detected	Truvada, Isentress, and Efavirenz	9.82	1	
07	37	White	Male	1230	1001	Not detected	Triumeq	6.70	3	
08	35	African American/Black	Male	559	869	34	Genvoya	4.51	2	Reversible biochemical hypothyroidism
09	50	African American/Black	Male	625	284	Not detected	Biktarvy	7.54	2	

**Fig. 1.**
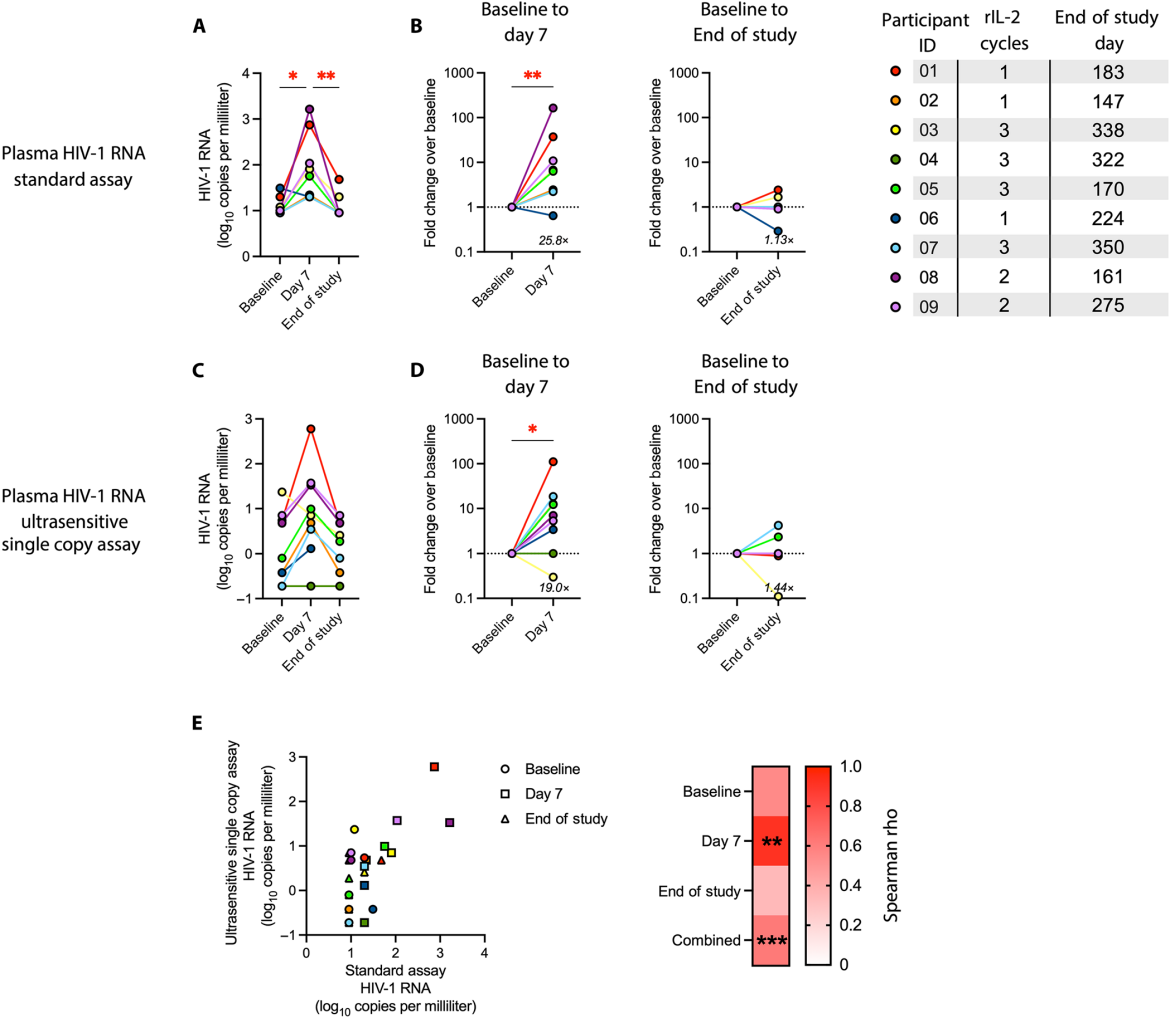
Virological outcomes of rIL-2 administration. Plasma HIV-1 RNA by standard clinical assay (**A**, **B**, and **E**) and by ultrasensitive single-copy assay (**C**, **D**, and E) at baseline, day 7 of rIL-2 treatment, and end of study shown as log_10_ copies per milliliter of plasma (A, C, and E) or fold change from baseline (B and D). (E) Correlation plot (left) and Spearman rho values (right) of HIV-1 RNA levels as detected by the two assays. Statistical significance was determined by Kruskal-Wallis test with Dunn’s correction for multiple comparisons (A and C), Wilcoxon matched-pairs signed rank test (B and D), or Spearman correlation (E). For Kruskal-Wallis tests, *adjusted *P* < 0.05; **adjusted *P* < 0.01; ***adjusted *P* < 001; for all others, asterisks indicate corresponding unadjusted *P* values. Only comparisons with *P* < 0.05 (or adjusted *P* < 0.05, when applicable) are indicated.

At day 7 (4 days after the last rIL-2 dose of the first treatment cycle), we found an average 26-fold increase in plasma HIV-1 RNA, compared to levels at baseline (an average of two time points: study entry and initiation of treatment), as measured by standard clinical assay (Fig. 1, A and B). Plasma HIV-1 levels returned to near baseline levels by the end of study [which, due to the premature discontinuation of the trial, was a different time point for each donor but was a median (IQR) of 23 (21 to 24.1) weeks after the initial rIL-2 administration and after a median of 2 (1 to 3) cycles of rIL-2]. Similar results were observed using an ultrasensitive single-copy plasma HIV-1 RNA assay: There was a 19.0-fold increase in plasma HIV-1 RNA at day 7 that returned to near baseline at end of study (Fig. 1, C and D). There was a strong correlation between HIV-1 RNA levels as measured in each assay, and this effect was driven mainly by the relationship at day 7, when HIV-1 RNA levels were most robust (Fig. 1E). Thus, rIL-2 is a potent inducer of latent HIV-1 reactivation in vivo in PWH on suppressive ART.

**Fig. 2.**
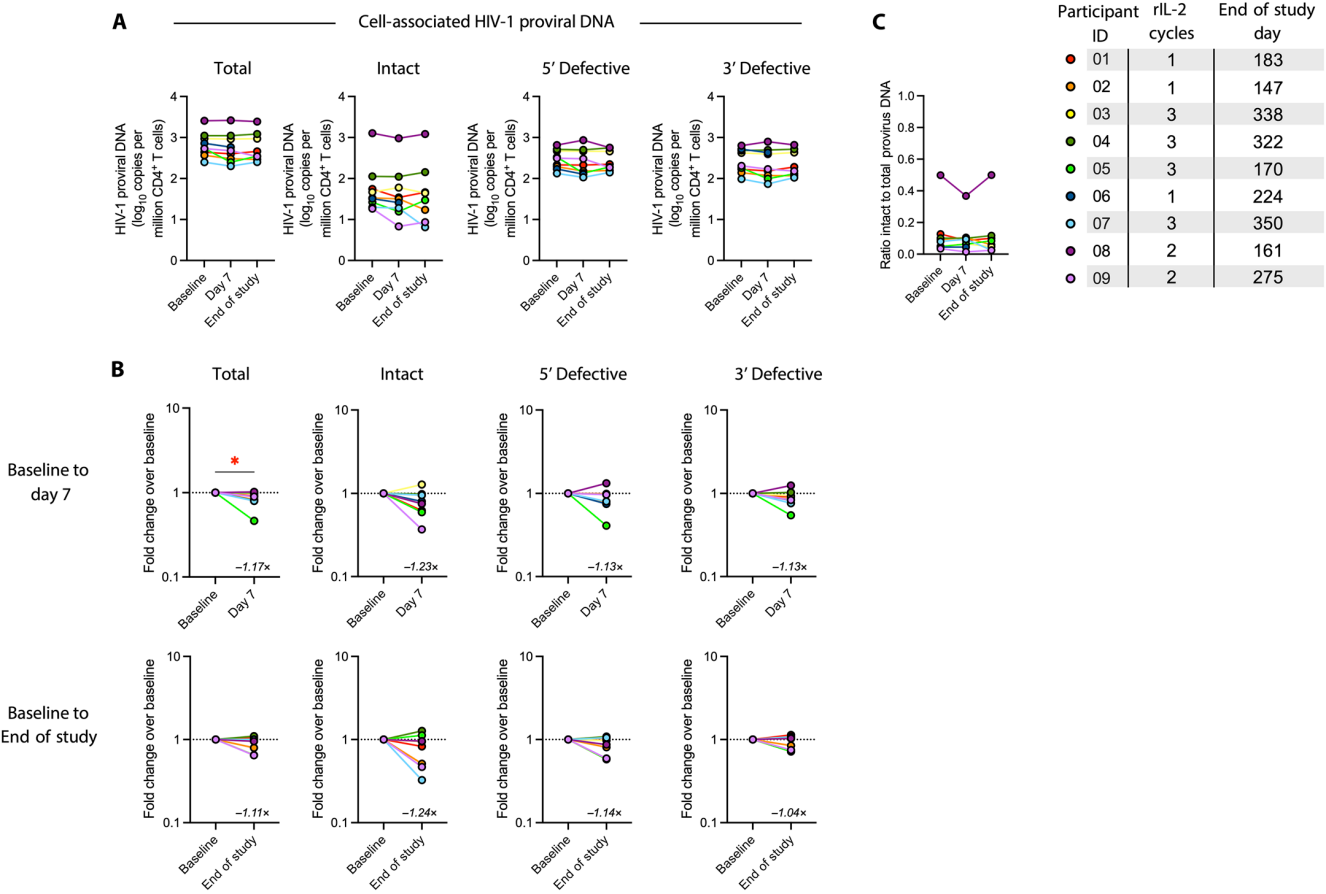
Effect of rIL-2 administration on the HIV-1 proviral reservoir. The amount of total, near full-length intact, 5′ defective, and 3′ defective cell–associated HIV-1 DNA proviruses in sorted CD4^+^ T cells at baseline, day 7 of rIL-2 treatment, and end of study as determined by IPDA shown as log_10_ copies per million CD4^+^ T cells (**A**) or fold change from baseline (**B**). (**C**) The ratio of intact to total HIV-1 DNA proviruses. Statistical significance was determined by Kruskal-Wallis test with Dunn’s correction for multiple comparisons (A and C) or by Wilcoxon matched-pairs signed rank test (B). *P < 0.05; only comparisons with *P* < 0.05 (or adjusted *P* < 0.05, when applicable) are indicated.

**Fig. 3.**
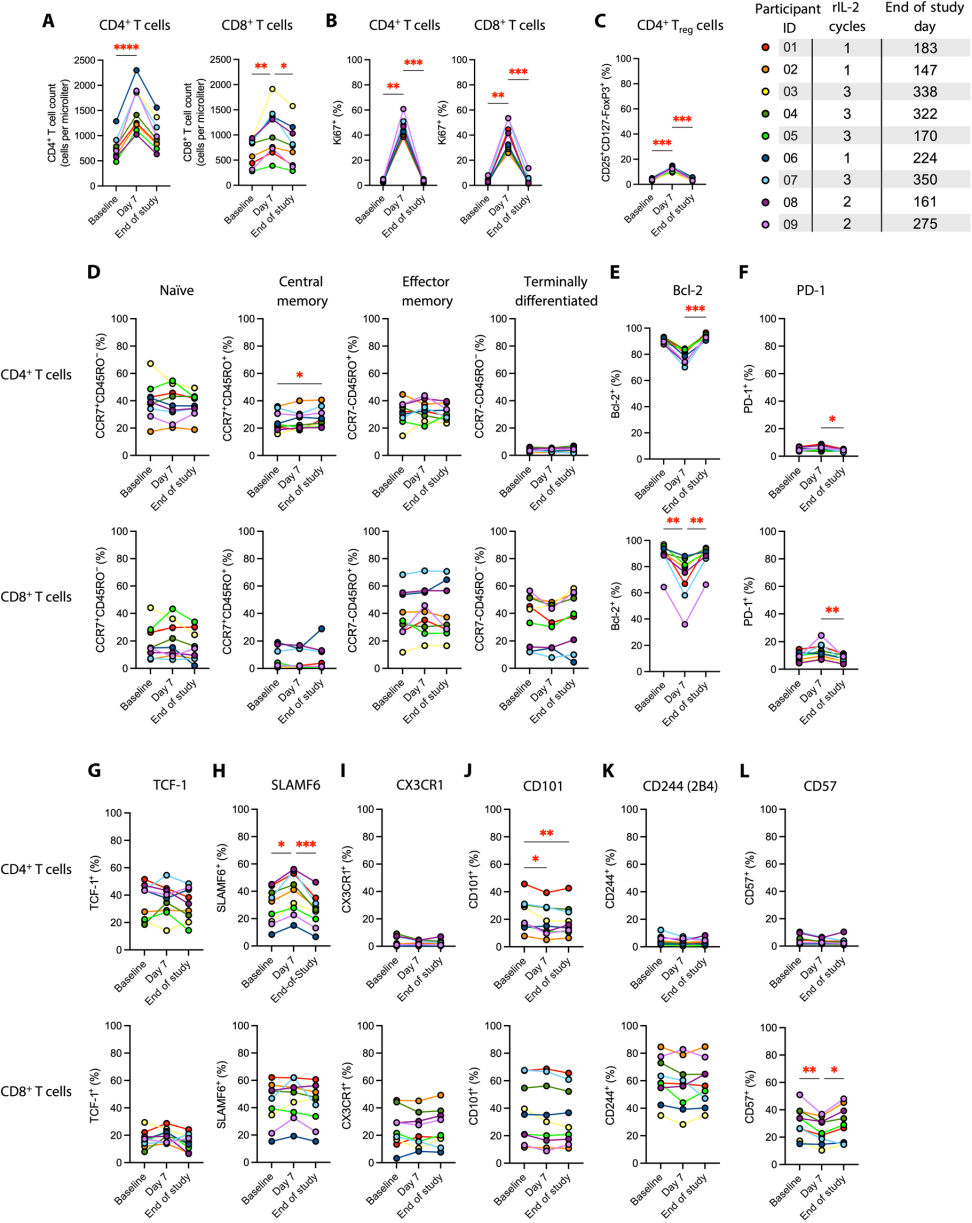
T cell outcomes of rIL-2 administration. (**A**) The number of CD4^+^ and CD8^+^ T cells/μl of peripheral blood at baseline, day 7 of rIL-2 treatment, and end of study. (**B**) The percent of CD4^+^ of CD8^+^ T cells that express Ki-67. (**C**) The proportion of CD4^+^ T cells that have a CD25^+^CD127-FoxP3+ T_reg_ phenotype. (**D** to **L**) The proportion of CD4^+^ (top rows) and CD8^+^ (bottom rows) T cells that are in each T memory compartment (D), or that express Bcl-2 (E), PD-1 (F), TCF-1 (G), SLAMF6 (H), CX3CR1 (I), CD101 (J), CD244/2B4 (K), or CD57 (L) at baseline, day 7 of rIL-2 treatment, and end of study. Statistical significance was determined by Kruskal-Wallis test with Dunn’s correction for multiple comparisons. *adjusted *P* < 0.05; **adjusted *P* < 0.01; ***adjusted *P* < 0.001; ****adjusted *P* < 0.0001. Only comparisons with adjusted *P* < 0.05 are indicated.

We next asked whether a single cycle of rIL-2 administration resulted in an effect on the HIV-1 reservoir. Using the intact provirus DNA assay (IPDA), we found a significant 1.2-fold reduction (i.e., a 20% decrease) in total HIV-1 proviral DNA copies per million CD4^+^ T cells and a trend toward reduced near full-length provirus sequences at day 7 (Fig. 2, A and B), with no effect on 5′ or 3′ defective proviral DNA sequences. As the number of near full-length provirus sequences returned to baseline levels by the end of study and the ratio of intact to total proviruses did not change (Fig. 2C), these results are likely due to a temporary dilutional effect by the expansion of cells lacking integrated HIV DNA, as the total number of circulating CD4^+^ T cells doubled (Fig. 3A).

**Fig. 4.**
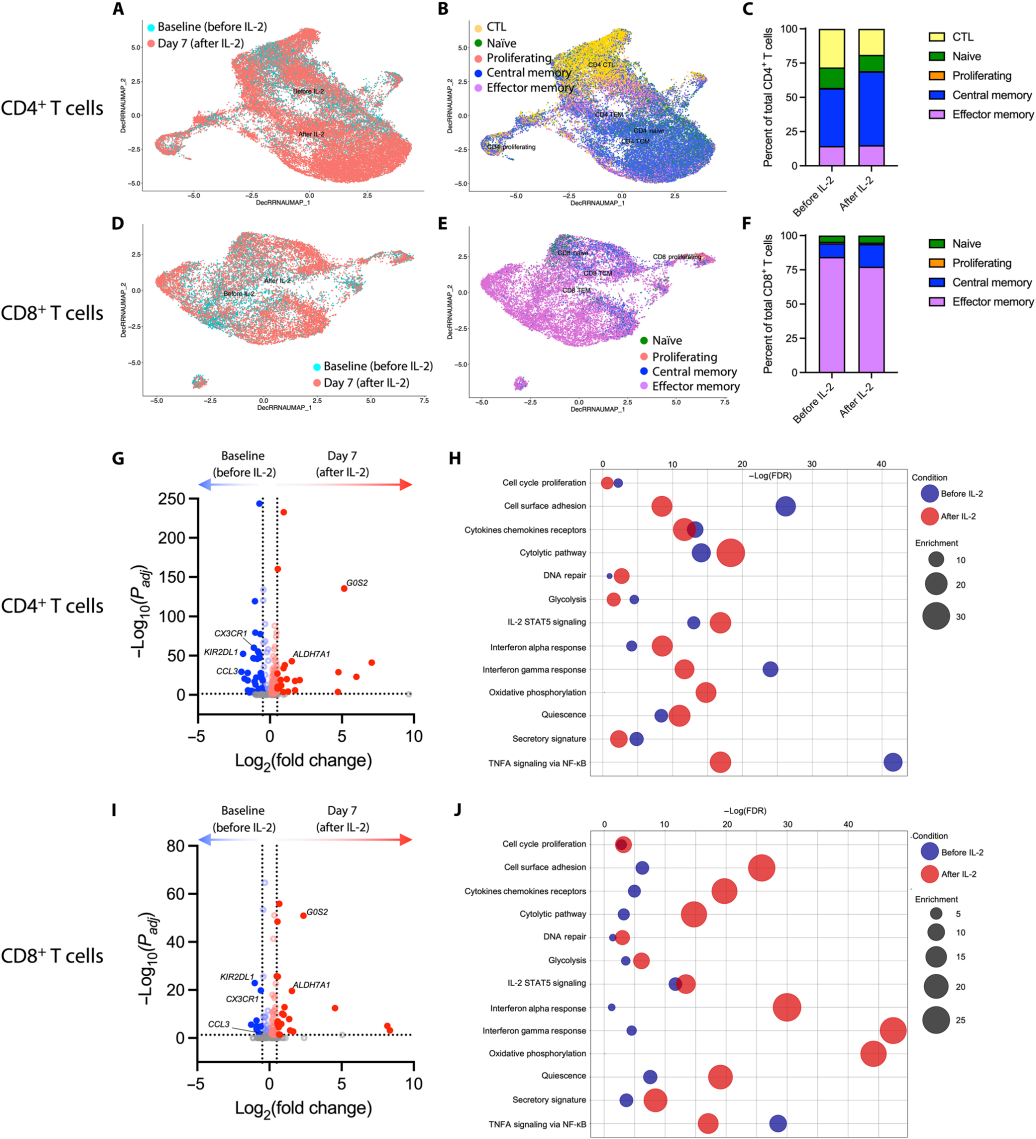
Single-cell RNA sequencing of T cells reveals transcriptional changes following rIL-2 administration. Single-cell RNA sequencing (AbSeq) analysis was performed on T cells from baseline or day 7 of rIL-2 treatment from four donors. (**A** to **F**) Integrated Uniform Manifold Approximation and Projection (UMAP) plots of denoised data show the shift in transcriptome (A and D) and phenotypic subsets (B and E) of CD4^+^ (A to C) and CD8^+^ (D to F) T cells due to rIL-2 administration. (C and F) Column graphs indicate the proportion of cells analyzed from baseline or day 7 (A and D) that are in the annotated subsets (B and E). (E to **H**) Volcano (E and G) and bubble (F and H) plots showing the differential gene expression and differential pathway enrichment, respectively, among CD4^+^ (E and F) and CD8^+^ (G and H) T cells. Annotated genes (**G** and **I**) are shared among T cells and NK cells.

The number of circulating CD8^+^ T cells increased 1.6-fold in the same period, and the percentage of CD4^+^ and CD8^+^ T cells that were in cell cycle, as evidenced by intracellular Ki67 expression, increased roughly 13-fold each from a median (IQR) of 3.75 (2.9 to 4.65) to 43.5 (42.2 to 48.3) for CD4^+^ T cells, and from 2.78 (2.14 to 3.42) to 32.6 (30.6 to 44.5) for CD8^+^ T cells at day 7 (Fig. 3, A and B). rIL-2 promotes CD4^+^ T regulatory (T_reg_) cell development, and we found a transient induction of the T_reg_ phenotype (CD25^+^CD127-FoxP3^+^) among CD4^+^ T cells (Fig. 3C). We found few changes in memory T cell subset proportions of either CD4^+^ or CD8^+^ T cells, apart from a persistent but subtle increase in CD4^+^ central memory T cells (Fig. 3D). Despite this memory subset stability, we observed robust phenotypic (Fig. 3, E to L) and transcriptional (Fig. 4, A to J) changes among CD4^+^ and CD8^+^ T cells following rIL-2 administration, with substantial up-regulation of interferon-α (IFN-α) response and oxidative phosphorylation pathways in both cell types (Fig. 4, H and J). Among key phenotypic changes at day 7 were a substantial drop in intracellular Bcl-2 expression in both CD4^+^ and CD8^+^ T cells, a significant increase in surface expression of SLAMF6, an activation and exhaustion marker, on CD4^+^ T cells, and a decrease in the exhaustion marker CD101 on CD4^+^ T cells (Fig. 3, E, H, and J). Among CD8^+^ T cells, expression of the senescence marker CD57 was significantly reduced (Fig. 3L). Although CD57 expression in CD8 T cells is associated with more cytolytic capacity (*21*), transcriptional analysis showed that cytolytic pathway components were considerably up-regulated following rIL-2 administration, consistent with a push toward enhancing the kill side of shock and kill. Other pathways, particularly cell surface adhesion, cytokines/chemokines/receptors, glycolysis, IFN-γ response, and secretory signature differed in their directionality, with CD4^+^ T cells exhibiting a transcriptional decrease in these pathways (Fig. 4H), and CD8^+^ T cells exhibiting transcriptional enhancement (Fig. 4J), after rIL-2 administration. Notably, we found a potent and consistent up-regulation of the cell cycling and metabolic genes *G0S2* and *ALDH7A1* among CD4^+^ and CD8^+^ T cells and NK cells (fig. S1A). Furthermore, *CCL3* (which encodes macrophage inflammatory protein–1α), *CX3CR1* (encoding the fractalkine receptor), and the killer cell immunoglobulin-like receptor gene *KIR2DL1* were consistently and robustly down-regulated at day 7 following rIL-2 administration among CD4^+^ and CD8^+^ T cells and NK cells (fig. S1B).

**Fig. 5.**
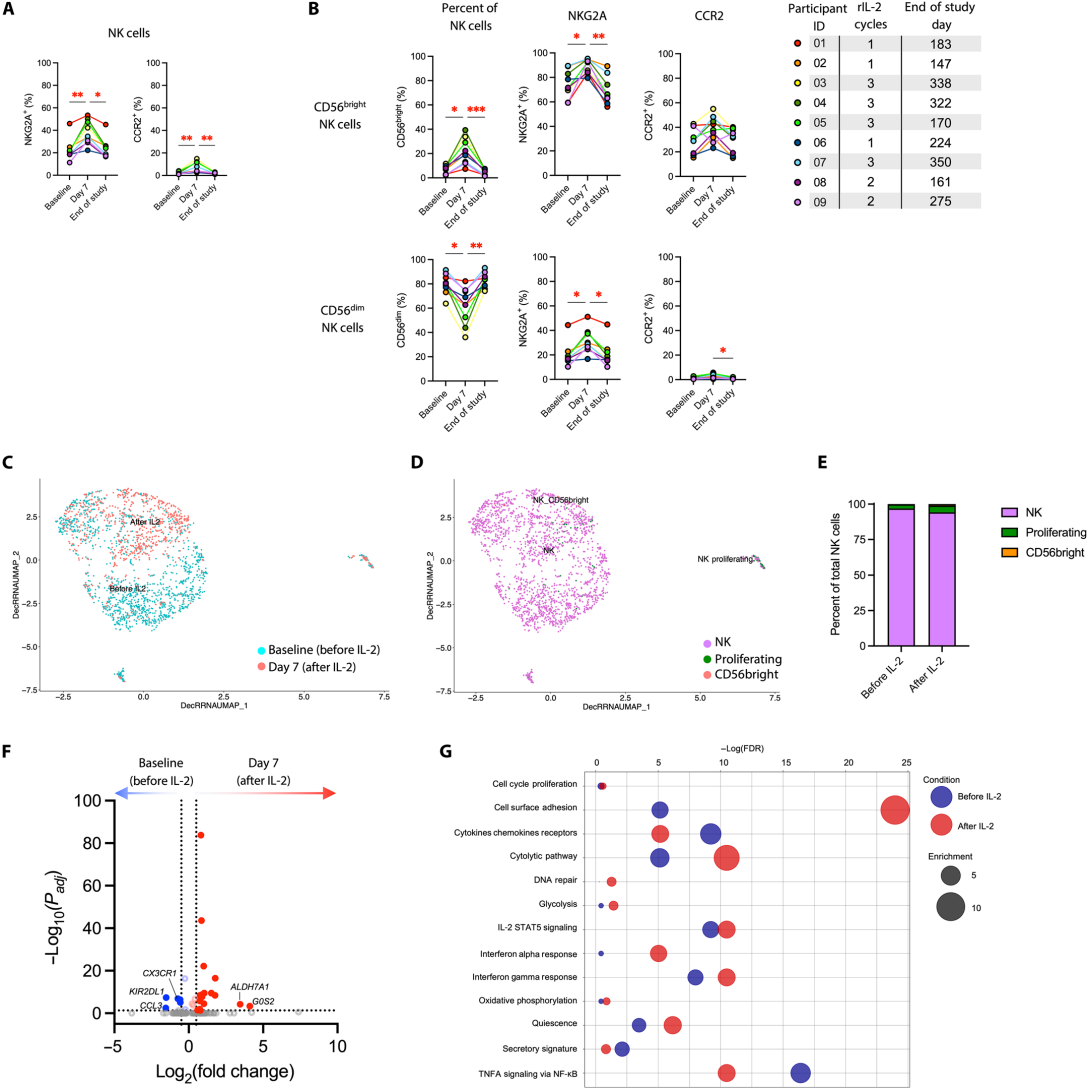
NK cell outcomes of rIL-2 administration. (**A**) The percent of NK cells that express NKG2A and CCR2. (**B**) The percent of NK cells that are CD56^bright^ (top) or CD56^dim^ (bottom), and the percent of CD56^bright^ or CD56^dim^ NK cells that express NKG2A or CCR2. (**C** to **G**) Single-cell RNA sequencing (AbSeq) analysis was performed on NK cells from baseline or day 7 of rIL-2 treatment from four donors. Integrated UMAP plots of denoised data show the shift in transcriptome (C) and phenotypic subsets (D) of NK cells due to rIL-2 administration. (E) Column graph indicating the proportion of cells analyzed from baseline or day 7 (C) that are in the annotated subsets (D). (F) Volcano plot showing the differential gene expression among NK cells. Annotated genes are shared among T cells and NK cells. (G) Bubble plot showing the differential pathway enrichment for NK cells. Statistical significance was determined by Kruskal-Wallis test with Dunn’s correction for multiple comparisons. *adjusted *P* < 0.05; **adjusted *P* < 0.01; ***adjusted *P* < 0.001. Only comparisons with adjusted *P* < 0.05 are indicated.

**Fig. 6.**
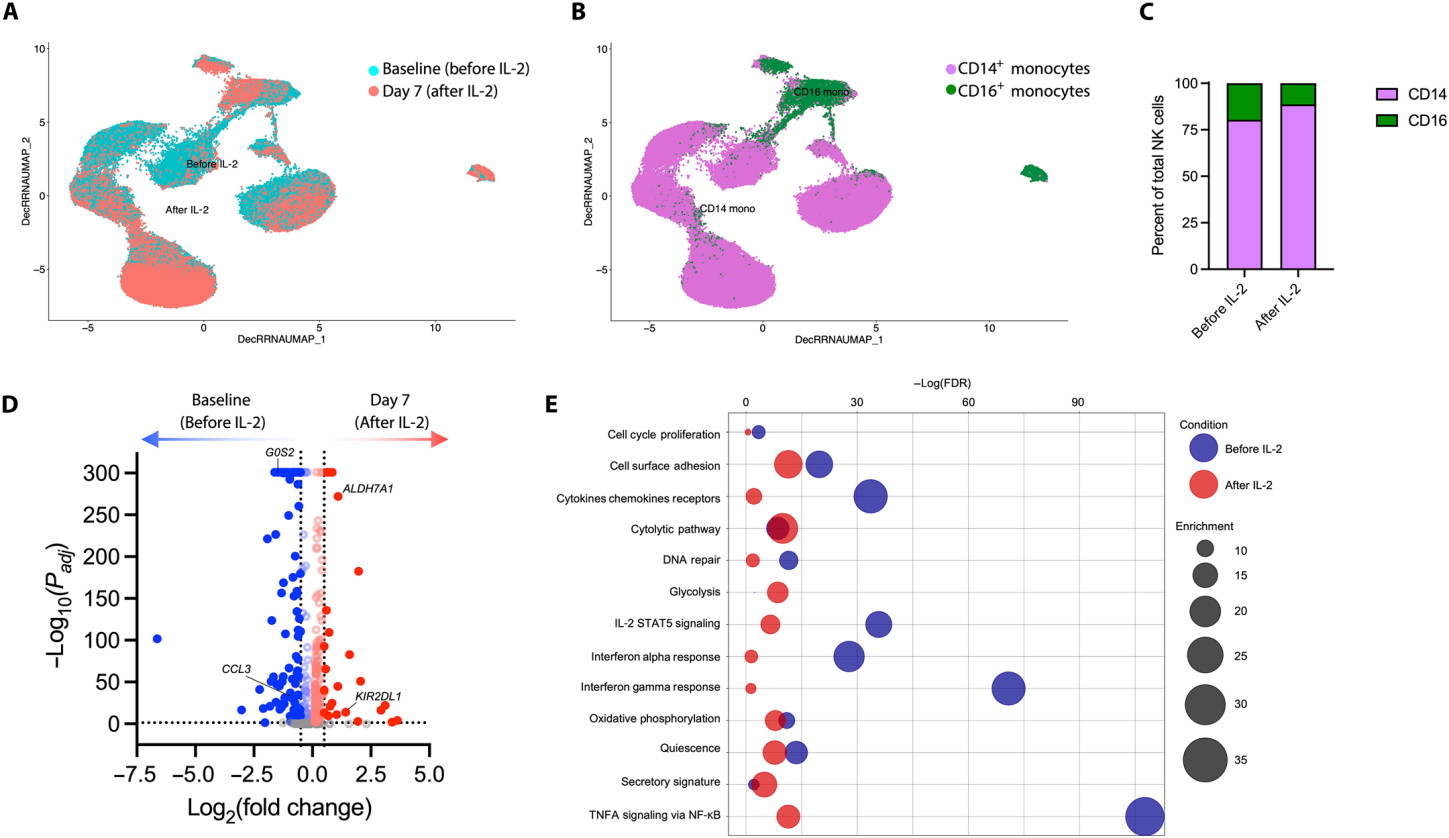
Monocyte outcomes of rIL-2 administration. Single-cell RNA sequencing (AbSeq) analysis was performed on monocytes from baseline or day 7 of rIL-2 treatment from four donors. Integrated UMAP plots of denoised data show the shift in transcriptome (**A**) and phenotypic subsets (**B**) of monocytes due to rIL-2 administration. (**C**) Column graph indicating the proportion of cells analyzed from baseline or day 7 (A) that are in the annotated subsets (B). (**D**) Volcano plot showing the differential gene expression among monocytes. Annotated genes are shared among monocytes, T cells, and NK cells. (**E**) Bubble plot showing the differential pathway enrichment for monocytes.

Previous trials have found that IL-2 increases NK cell activity (*22*–*25*). Human NK cells can broadly be separated into two major subsets based on CD56 expression. CD56^bright^ NK cells express the high-affinity IL-2 receptor and are immunomodulatory cytokine producers, while CD56^dim^ NK cells are largely cytotoxic and elicit antibody-dependent cellular cytotoxicity (*26*). Here, we found that rIL-2 administration induced significant surface up-regulation of the human leukocyte antigen–E–binding inhibitory receptor NKG2A and the monocyte chemotattractant protein–1 (MCP-1/CCL2) receptor CCR2 on circulating NK cells (Fig. 5A). The proportion of NK cells that were CD56^bright^ increased significantly following rIL-2 administration, as did NKG2A expression, independent of whether the cells were CD56^bright^ or CD56^dim^ (Fig. 5B). We also observed that rIL-2 administration promoted transcriptional changes in NK cells (Fig. 5, C to G), eliciting mostly similar effector and metabolic pathways in NK cells as induced in CD8^+^ T cells (Fig. 5G), although the robust expansion of CD56^bright^ NK cells was not detected in the transcriptional data (Fig. 5, D and E). Conversely, the transcriptional milieu of circulating monocytes was mostly down-regulated, with decreased expression of pathways related to tumor necrosis factor (TNF) signaling and responses to IFN-α and IFN-γ (Fig. 6, A to E). In contrast to the transcriptional patterns among lymphocytes, *G0S2* expression was significantly down-regulated, and *KIR2DL1* expression was significantly up-regulated, in monocytes following rIL-2 administration (Fig. 6D).

**Fig. 7.**
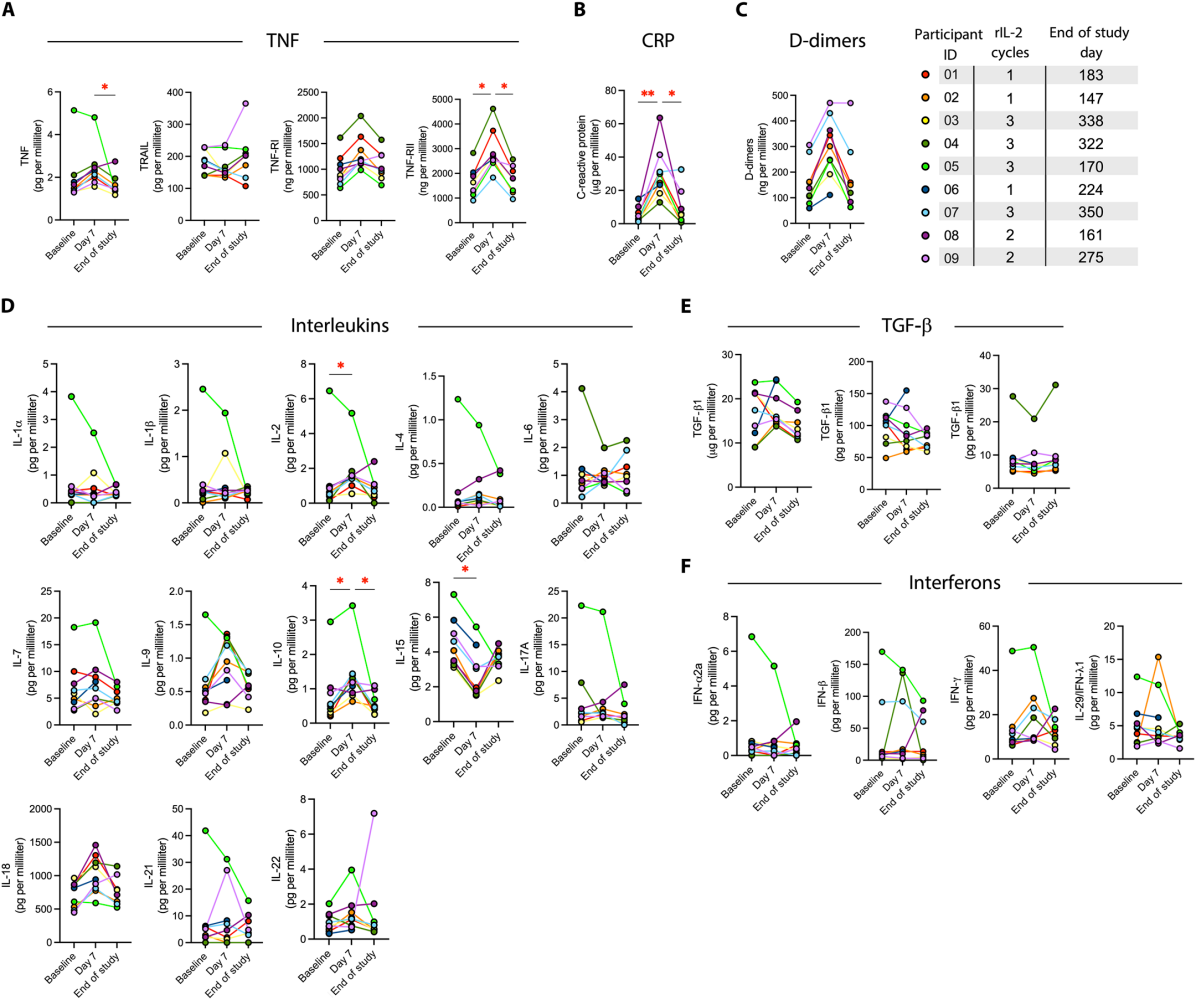
Effect of rIL-2 administration on soluble plasma analytes. Soluble levels of (**A**) TNF, TNF-related apoptosis-inducing ligand (TRAIL), and TNF receptors; (**B**) CRP; (**C**) D-dimers; (**D**) interleukins; (**E**) tumor growth factor–β (TGF-β) proteins; and (**F**) IFNs were measured in plasma by enzyme-linked immunosorbent assay (TNF-RI, TNF-RII, and D-dimers) or Mesoscale Discovery (all others) at baseline, day 7 of rIL-2 treatment, and end of study. Statistical significance was determined by Kruskal-Wallis test with Dunn’s correction for multiple comparisons. *adjusted *P* < 0.05; **adjusted *P* < 0.01. Only comparisons with adjusted *P* < 0.05 are indicated.

We next investigated the effects of rIL-2 on the systemic inflammatory response. Overall, we found high variability with few consistent patterns (Fig. 7, A to F), apart from a general increase in inflammation, as evidenced by significantly increased TNF-RII and C-reactive protein (CRP) levels (Fig. 7, A and B) and increases in D-dimers and IL-18 that did not reach statistical significance (Fig. 7, C and D); all of these returned to baseline levels by the end of the study. Levels of the immunoregulatory cytokine IL-10 also increased, consistent with a compensatory counter-inflammatory response (*27*). Furthermore, we detected a significant increase in levels of IL-2, but whether these levels were due to residual rIL-2 or to de novo IL-2 synthesis is unclear. Levels of IL-15 were significantly reduced following IL-2 administration and remained lower through the end of the study (Fig. 7D). Like IL-2, IL-15 is a common γ-chain cytokine that also uses the IL-2Rβ subunit, and the capacity of the rIL-15 superagonist N-803 has been tested for its capacity to elicit HIV reactivation from latency in vivo with modest results (*28*).

## DISCUSSION

A variety of agents can induce HIV-1 reactivation in vitro from CD4^+^ T cells obtained from PWH (*7*), some of which have been tested in vivo for their capacity to reactivate latent virus or to reduce the viral reservoir. Exogenous administration of IL-2 is one such agent among the shock and kill strategies with the potential to reduce the viral reservoir, as it should elicit the shock by promoting HIV-1 expression and enhance the kill by triggering T cell and NK cell activation. Here, we found that rIL-2 administration potently reversed HIV-1 latency and elicited robust NK and T cell activation, exemplified by phenotypic and transcriptional alterations in cell metabolism and effector functions. Despite these effects, there was no clear reduction of intact HIV-1 proviruses in peripheral blood CD4^+^ T cells after just one cycle of IL-2 administration, suggesting that a substantial and sustained reduction of the HIV-1 reservoir will require multiple cycles, or use of IL-2 in combination with other approaches.

The donor with the highest HIV-1 RNA levels at baseline in each assay exhibited reduced HIV-1 RNA levels at day 7, which was further decreased at the end-of-study measurement. Given the low numbers of participants in our study, we cannot draw too strong a conclusion from these findings, but they may suggest that the efficacy and/or the mechanism of rIL-2 administration–induced reactivation may differ between individuals with and without active viral replication.

We observed substantial phenotypic and transcriptional changes among immune cells at day 7 following rIL-2 administration. Among CD4^+^ T cells, SLAMF6 was significantly up-regulated, and Bcl-2 and CD101 were significantly down-regulated. Surface expression of TCF-1, CX3CR1, CD244, and CD57 did not change. Among CD8^+^ T cells, rIL-2 induced significant reductions in Bcl-2 and CD57 but no changes among the other markers. Intriguingly, for both CD4^+^ and CD8^+^ T cells, expression of the inhibitory receptor PD-1 was significantly reduced at the end-of-study time points, perhaps indicating a long-term effect of rIL-2 administration. Among NK cells, we saw significant increases in NKG2A and CCR2 expression, as well as in the proportion of CD56^bright^ NK cells. Although CD56^bright^ NK cells are usually considered less cytotoxic and more immunoregulatory, we observed robust up-regulation of the cytolysis transcriptional pathway both in CD8^+^ T cells and in NK cells.

Our study leaves some unanswered questions, such as the tissue and cellular sources of the HIV-1 plasma RNA and whether the concurrent induction of CD4^+^ T_reg_ cells impairs the capacity of the activated immune cells to kill infected CD4^+^ T cells. Knowing where in the body the latent virus reservoir is maintained, and which reservoir cells are the most likely to reactivate during latency-reversal treatment or analytic treatment interruption, are key factors in understanding how best to facilitate HIV-1 cure strategies. Recent work in monkeys and humans has elucidated the complexity of viral reservoirs, suggesting that viruses from multiple anatomical sites contribute to plasma viremia, but that these viruses reach new tissues via the bloodstream (*29*–*31*). Thus, the viruses we detect in the blood are unlikely to originate from circulating CD4^+^ T cells but rather from cells within tissues, such as lymphoid germinal centers, where they may be partially or wholly inaccessible to immune targeting by cytolytic CD8^+^ T cells and/or NK cells (*32*). There is a large variation in the capacity of LRAs to induce HIV-1 reactivation, with up to a 100-fold difference in the fold induction of HIV-1 transcription both among LRA classes but also between individuals who are exposed to the same LRA (*33*). The site of HIV-1 integration into the host genome can also determine the extent of cellular activation and HIV-1 expression after LRA exposure (*34*–*36*). The distribution of proviral integration sites could explain the variation in rIL-2–mediated viral reactivation seen among study participants. While this initial report is mostly focused on immunological changes associated with rIL-2 administration, we hope to follow up our studies with more extensive virological analyses.

CD4^+^ T_reg_ cells have heightened sensitivity to IL-2, allowing for a competitive advantage for low-dose IL-2, and numerous trials have attempted to alleviate autoimmune disorders by increasing T_reg_ cells with low-dose rIL-2 administration with varying levels of success (*37*). Whether the T_reg_ cells elicited by rIL-2 here restrict the targeting of HIV-expressing CD4^+^ T cells is currently unknown and additional experiments are needed to explore the relationship between these effects of rIL-2 administration on activation of T_reg_ cells and other T cells and the reactivation and persistence of HIV reservoirs.

Despite initiating both the shock and the kill, IL-2 monotherapy is likely insufficient as a curative strategy, and the application of more sustained lower, tolerable doses of IL-2 or combination strategies might provide more successful means to target the HIV-1 reservoir in PWH well-controlled with ART. Because of its antitumor efficacy, IL-2 has been a particular focus for bioengineering strategies. Constructs of rIL-2 that bypass CD25 engagement to avoid or reduce T_reg_ activity, such as IL-2 superkines or polyethylene glycol (PEG)ylated formulations, could activate effector responses without eliciting compensatory inhibitory pathways (*38*–*40*). Another strategy could use orthogonal (*ortho*) receptor-ligand pairs engineered to exclusively signal in combination. For instance, PWH could receive adoptive cell therapy with effector cells specific for HIV-1 (either naturally or engineered) that express an *ortho*IL-2Rβ construct that is unable to bind native IL-2 in conjunction with an *ortho*IL-2 molecule that selectively binds *ortho*IL-2Rβ (*41*). In this setting, only the transferred cells that express *ortho*IL-2Rβ will expand, limiting the “off-target” T_reg_ induction. Immunocytokines are another potential strategy. Immunocytokines are constructs that use antibody architecture to deliver cytokine signals in *cis* or in close proximity to the antibody target to geographically limit their effects (*39*, *40*). PD1-IL2v, for example, has been shown to deliver IL-2 signals in *cis* to IL-2Rβγ–expressing cells that also express PD-1 (*42*). If a suitable surface molecule that identifies HIV-infected T cells is identified, then that molecule could be coupled to IL-2 in an immunocytokine formulation to deliver IL-2 signaling to nearby CD8^+^ T cells and NK cells. Last, cytokine adaptors—soluble molecules that convert one signaling input into another—could be used to redirect immunosuppressive signals [such as IL-10 or transforming growth factor–β (TGF-β)] into IL-2 signals (*43*). Thus, the development of these and other modalities that might strengthen the antiviral properties of IL-2, while limiting IL-2–associated toxicity, leaves the door open for keeping rIL-2 in the arsenal to fight HIV-1.

## MATERIALS AND METHODS

### Participants and study design

Participants were enrolled after providing written informed consent between 02 April 2019 and 16 August 2019 at University Hospitals Cleveland Medical Center (UHCMC) under UHCMC institutional review board approval # 20180025. This trial is registered at clinicaltrials.gov # NCT03308786. Eligibility in this open-label, single-arm trial of rIL-2 administration included documented HIV-1 infection controlled by stable ART with undetectable viral loads for at least 1 year allowing for intermittent isolated episodes of detectable viremia <500 copies RNA/ml, age 18 to 65 years, and CD4^+^ T cell counts greater than 350 cells/μl. Acute or chronic hepatitis C virus or hepatitis B virus infections, history of advanced liver or lung diseases, and childbearing potential for female participants were among the exclusion criteria. Participants were to self-administer rIL-2 (aldesleukin, Proleukin, Prometheus Laboratories) subcutaneously at an initial dose of approximately 5 MIU twice daily for 4 consecutive days every 8 weeks (for a total of eight cycles). All end points in this report were measured at day 7 of the first cycle (3 days after the last dose of treatment) and at the last time point collected (end of study), which differs for each participant.

### Plasma HIV-1 RNA measurement

Plasma levels of HIV-1 RNA were measured in the clinic using the Cobas 6800/8800 system (Roche). Ultrasensitive single-copy assay was performed by Accelevir Diagnostics.

### Cellular HIV-1 proviral DNA measurement

CD4^+^ T cells were sorted from cryopreserved peripheral blood mononuclear cells (PBMCs), and cellular HIV-1 proviral DNA was sequenced from using the IPDA by Accelevir Diagnostics.

### Flow cytometry

Cryopreserved PBMC specimens were thawed, washed, and separated into three aliquots, and each aliquot was stained with LiveDead Aqua viability dye and one of three comprehensive panel of antibodies. For analysis of conventional T cell cycling: fluorochrome-conjugated antibodies targeting CD3 (clone UCHT1, BUV737, BD Biosciences), CD4 (clone SK3, BUV395, BD Biosciences), CD8 (clone SK1, BV605, BD Biosciences), CD45RO (clone UCHL1, BV650, BD Biosciences), CCR7 (clone 2-L1-A, APC-R700, BD Biosciences), FoxP3 [clone 259D/C7, phycoerythrin (PE), BD Biosciences], CD25 (clone BC96, BV711, BD Biosciences), CD127 (clone A019D5, PE-Dazzle594, BioLegend), PD-1 (clone EH12.1, BV786, BD Biosciences), Ki-67 (clone B56, PE-Cy7, BD Biosciences), Bcl-2 [clone Bcl-2/100, fluorescein isothiocyanate (FITC), BD Biosciences], CD57 [clone HNK-1, allophycocyanin (APC), BioLegend], TIM-3 (clone 7D3, PerCP-Cy5.5, BD Biosciences), and TCF-1 (clone S33-966, BV421, BD Biosciences). For analysis of NK cells and nonconventional T cells: fluorochrome-conjugated antibodies targeting CD3 (clone UCHT1, BUV737, BD Biosciences), CD4 (clone SK3, BUV395, BD Biosciences), CD8 (clone SK1, BV605, BD Biosciences), CD45RO (clone UCHL1, BV650, BD Biosciences), CCR7 (clone 2-L1-A, APC-R700, BD Biosciences), CD25 (clone BC96, BV711, BD Biosciences), CD127 (clone A019D5, PE-Dazzle594, BioLegend), PD-1 (clone EH12.1, BV786, BD Biosciences), CD161 (clone DX12, APC, BD Biosciences), CCR2 (clone K036C2, BV421, BioLegend), CD16 (clone B73.1, PE-Cy5, BioLegend), CD56 (clone MEM-188, AF488, BioLegend), NKG2A (clone S19004C, PE, BioLegend), and T cell receptor Vα7.2 (clone 3C10, PE-Cy7, BioLegend). For analysis of T cell immune checkpoint molecules: fluorochrome-conjugated antibodies targeting CD3 (clone UCHT1, BUV737, BD Biosciences), CD4 (clone SK3, BUV395, BD Biosciences), CD8 (clone SK1, BV605, BD Biosciences), CD45RO (clone UCHL1, BV650, BD Biosciences), CCR7 (clone 2-L1-A, APC-R700, BD Biosciences), CD25 (clone BC96, BV711, BD Biosciences), CD28 (clone CD28.2, PE-Dazzle594, BioLegend), PD-1 (clone EH12.1, BV786, BD Biosciences), TIM-3 (clone 7D3, PerCP-Cy5.5, BD Biosciences), CD57 (clone HNK-1, FITC, BioLegend), CD101 (clone BB27, PE-Cy7, BioLegend), CD244/2B4 (clone C1.7, BV421, BioLegend), CX3CR1 (clone 2A9-1, APC, BioLegend), and SLAMF6 (clone hSF6.4.20, PE, BD Biosciences). Surface stains were performed at room temperature for 20 min. Intracellular stains were performed for 40 min on ice following 20 min of fixation and permeabilization with the eBioscience FoxP3 staining kit. All samples were then fixed in 1% paraformaldehyde for 15 min at room temperature before a final wash and resuspension in buffer for acquisition on an LSRFortessa cytometer (BD Biosciences).

### Soluble analytes

Plasmas were analyzed in batch per the manufacturer’s protocols by enzyme-linked immunosorbent assay for levels of D-dimers (Asserachrom), TNFR-I (R&D Systems), or TNFR-II (R&D Systems), or by Mesoscale Discovery U-plex for levels of IFN-α2a, IFN-β, IFN-γ, IL1α, IL1β, IL-2, IL-4, IL-7, IL-9, IL-10, IL-15, IL-18, IL-21, IL-22, IL-29/IFN-λ1, TNF, TRAIL, TGF-β-I, TGF-β-II, TGF-β-III (Mesoscale Diagnostics), S-plex for levels of IL-6 or IL-17A, and V-plex for levels of CRP.

### scRNA sequencing

PBMCs (~500,000 cells per sample) were stained with the BD Immune Discovery Panel of 30 oligonucleotide-conjugated antibodies supplemented with an additional pool of 52 antibodies including the functional markers for T cell subsets (Supplementary table, fasta file of AbSeq barcodes). Single-cell RNA sequencing (scRNA-seq)/Abseq was performed using the BD Rhapsody Express platform. Whole transcriptome and AbSeq libraries were sequenced at Medgenome Inc., using Illumina HiSeq X, PE150. Fastq files were mapped against the GRCh38 human genome reference and AbSeq barcodes. Count tables were analyzed using Seurat package for R (*44*). Single-cell profiles with a minimum of 500 genes per cell, containing less than 25% mitochondrial mRNA and predicted doublets removed [DoubletFinder package for R (*45*)] resulting in a total of ~275,000 scRNA-Seq/AbSeq profiles for the eight samples (four participants, two samples per participant). Denoising of RNA and protein counts were performed using DecontX package for R (*46*). We used Seurat for gene count normalization, scaling, identifying variable genes, principal component and cluster analysis, and Uniform Manifold Approximation and Projection (UMAP) dimensionality reduction. Cell types were predicted by computational mapping to a reference PBMC dataset (*47*).

### Statistical analysis

Most comparisons included baseline, an average of two time points: study entry and initiation of treatment; day 7, which was 3 days after the last rIL-2 dose; and end of study, which due to the premature discontinuation of the trial, was a different time point for each donor, but was a median (IQR) of 23 (21 to 24.1) weeks after the initial rIL-2 administration and after a median of 2 (1 to 3) cycles of rIL-2. We used Kruskal-Wallis tests with Dunn’s correction for multiple comparisons, and an adjusted *P* value is shown. Fold change analyses were compared with Wilcoxon matched-pairs signed rank test. Correlations were compared by Spearman analysis. Upstream transcription factor prediction was performed using the Transcriptional Regulatory Relationship Unraveled by Sentence-based Text mining (TRRUST v2) online database (*48*). Protein-protein interactions were predicted and visualized using the STRING (v12.0) online database (*49*). All analyses were performed using GraphPad Prism v10.6.
