# Supplementary material for: Interleukin-2 is a potent latency reversal agent in people with treated HIV-1
Source: Sci Adv. 2025 Dec 19;11(51):eaea4268. doi: 10.1126/sciadv.aea4268 (PMC12716389; doi:10.1126/sciadv.aea4268)
Supplement: Supplementary file 1 — Fig. S1 [file sciadv.aea4268_sm.pdf]

Science Advances
AAAS

Supplementary Materials for

Interleukin-2 is a potent latency reversal agent in people with treated HIV-1

Michael L. Freeman *et al.*

Corresponding author: Michael L. Freeman, mlf62@case.edu;
Michael M. Lederman, lederman.michael@clevelandactu.org

Sci. Adv. **11**, eaea4268 (2025)
DOI: 10.1126/sciadv.aea4268

This PDF file includes:

Fig. S1

A

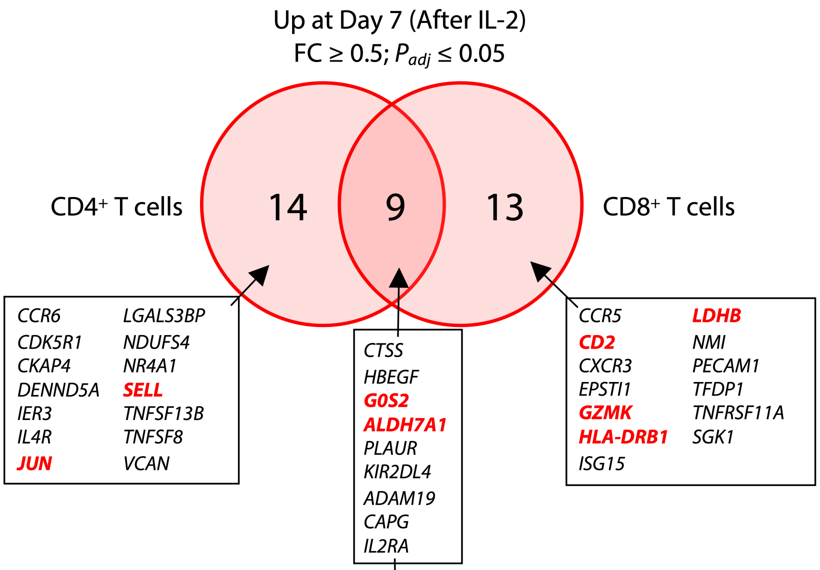

Key TF	*FDR*
RELA	0.0006
NFKB1	0.0006
SP3	0.0017

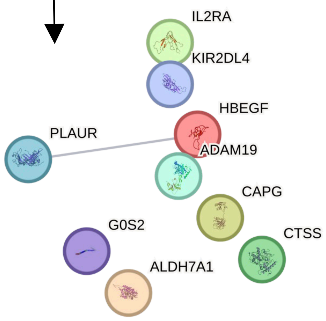

B

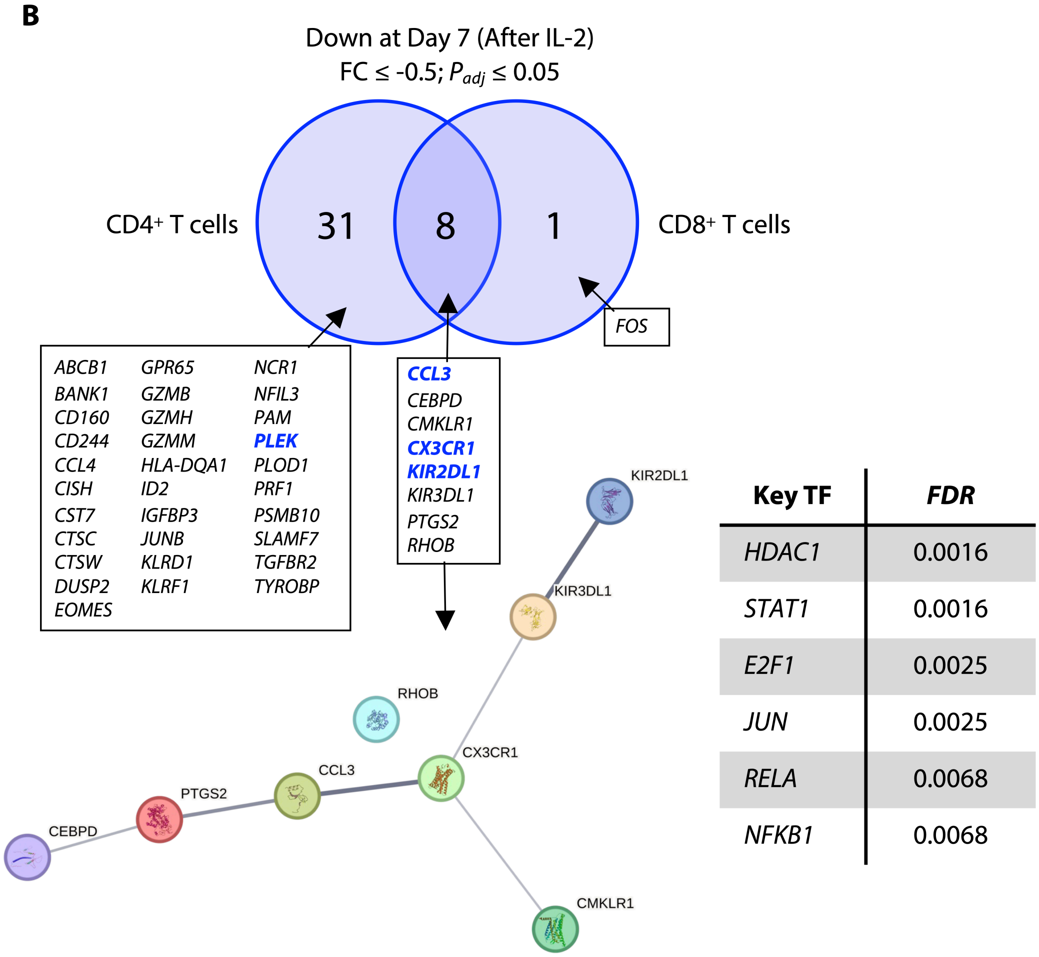

Key TF	*FDR*
HDAC1	0.0016
STAT1	0.0016
E2F1	0.0025
JUN	0.0025
RELA	0.0068
NFKB1	0.0068

Fig. S1. T cell transcriptional signatures of recombinant IL-2 administration. Single-cell RNA-sequencing (AbSeq) analysis was performed on T cells from baseline or day 7 of rIL-2 treatment from 4 donors. **(A)** (*top*) Genes that are significantly ($P_{adj} \leq 0.05$) and substantially (log_2(fold change) ≥ 0.5) upregulated at day 7 vs. baseline in CD4+ and CD8+ T cells. Bolded red genes are shared among T cells and NK cells. (*bottom*) Key upstream transcription factors (TF) and false discovery rate (FDR), as determined by TRRUST database analysis, and network map, as determined by STRING database analysis, of the shared upregulated genes. **(B)** (*top*) Genes that are significantly ($P_{adj} \leq 0.05$) and substantially (log_2(fold change) ≥ -0.5) downregulated at day 7 vs. baseline in CD4+ and CD8+ T cells. Bolded blue genes are shared among T cells and NK cells. (*bottom*) Key upstream TF and FDR driving expression, as determined by TRRUST database analysis, and network map, as determined by STRING database analysis, of the shared downregulated genes.
